# Risk Stratification for Sudden Cardiac Death In Patients With Non-ischemic Dilated Cardiomyopathy

**Published:** 2005-04-01

**Authors:** Karthik Shekha, Joydeep Ghosh, Deepak Thekkoott, Yisachar Greenberg

**Affiliations:** Department of Cardiology and Clinical Electrophysiology, Maimonides Medical Center (Mount Sinai Health System), Brooklyn, New York.

**Keywords:** Non Ischemic Dilated Cardiomyopathy, Ischemic Dilated Cardiomyopathy, Risk Stratification, Implantable cardioverter defibrillator, Sudden Cardiac Death

## Abstract

Non ischemic dilated cardiomyopathy (NIDCM) is a disorder of myocardium. It has varying etiologies. Albeit the varying etiologies of this heart muscle disorder, it presents with symptoms of heart failure, and rarely as sudden cardiac death (SCD). Manifestations of this disorder are in many ways similar to its counterpart, ischemic dilated cardiomyopathy (IDCM). A proportion of patients with NIDCM carries a grave prognosis and is prone to sudden cardiac death from sustained ventricular arrhythmias. Identification of this subgroup of patients who carry the risk of sudden cardiac death despite adequate medical management is a challenge .Yet another method is a blanket treatment of patients with this disorder with anti arrhythmic medications or anti tachyarrhythmia devices like implantable cardioverter defibrillators (ICD). However this modality of treatment could be a costly exercise even for affluent economies. In this review we try to analyze the existing data of risk stratification of NIDCM and its clinical implications in practice.

Non-ischemic dilated cardiomyopathy (NIDCM) is a primary disease of the myocardium, characterized by dilatation of all four chambers of the heart, but primarily the left ventricle, with associated systolic dysfunction. The incidence of dilated cardiomyopathy is 5 - 8 / 100,000 / year [1,2]. In an international heart failure study it was found that 18% of symptomatic patients with ejection fraction less than 30% were diagnosed with NIDCM[3]. The age-adjusted mortality in patients with NIDCM ranges from 0.10 per 10,000 person-years among men aged 35-39 up to 1.16 per 10,000 person-years among men aged 55-57 [2]. Currently, mortality in NIDCM is 12-13% at 3 years [4]. Independent risk factors for death include smoking, diabetes mellitus, and high diastolic blood pressure [2]. Patients with NIDCM suffer from heart failure mortality and sudden cardiac death in near equal numbers [5]. Sudden cardiac death (SCD) may well be the first manifestation of NIDCM, and idiopathic NIDCM is responsible for 10% of all sudden cardiac deaths in adults [6]. Survivors of SCD with NIDCM often have recurrent ventricular fibrillation as their clinical arrhythmia [7].

The majority of SCDs occur among patients that are classically defined as low risk, including those with NYHA functional class II or I. In the MERIT-HF trial (NYHA heart failure class II-IV, EF < 40%), the most common cause of mortality in patients with NYHA functional classes II and III was sudden cardiac death[8]. The incidence of SCD in NYHA class IV heart failure is also high but the competing risk of pump failure makes it the second cause of death in this very sick category of patients.

With the advent of the implantable cardioverter defibrillator (ICD) and their near-perfect termination of lethal arrhythmias, a determination needs to be made of which patients would benefit most from ICD insertion. In order to balance the potential risks of device implantation and the associated cost, many investigators have sought to establish risk factors to select patients with NIDCM who would benefit most from ICD implantation.

## Secondary Prevention Trials

Patients with symptomatic NIDCM and documented ventricular tachycardia or ventricular fibrillation (VT/VF) have a mortality rate of up to 22% in the first two years after diagnosis. This is comparable to a similar group of patients in the AVID registry with CAD[9]. It has been clearly established that patients with symptomatic sustained ventricular tachycardia and underlying structural heart disease benefit from ICD implantation irrespective of the etiology of their heart disease [10-21]. Data from the AVID registry reveal a similar mortality rate in patient’s surviving symptomatic VT or VF regardless of the underlying nature of cardiac disease. The AVID trial, on the other hand, randomized patients resuscitated from ventricular arrhythmias to either ICD therapy or treatment with class III antiarrhythmic agents (primarily amiodarone) [22]. Patients were randomized (n=1016) and 3117 patients were followed in the AVID registry. The registry comprised 73% of the patients with CAD, while the remaining 27% were diagnosed as NIDCM.  54% of NIDCM patients received ICDs, while 48% of CAD patients received ICDs. Analysis of the registry revealed that the overall 2-year survival of patients with NIDCM was 78.2%, which was similar to those with CAD.

## Primary Prevention

Primary prevention of sudden cardiac death is an important goal in the NIDCM population. Two trials using amiodarone in a randomized fashion against placebo have been performed in the NIDCM population. The GESICA trial randomized 516 patients with predominantly non-ischemic cardiomyopathies and ejection fraction <35% on optimal medical therapy to amiodarone or placebo [23]. The trial demonstrated a substantial reduction in all cause mortality in patients treated with amiodarone (33.5% vs. 41.4% RR reduction 28%, 95% CI 4-45%) independent of the presence of ventricular arrhythmias. Although the trial was randomized, it was criticized for its non-blinded enrollment. Also many patients enrolled in this trial had cardiomyopathy from Chaga’s disease, which is a very different group of patients compared to NIDCM patients in the US or other parts of the world.

Similarly, CHF-STAT evaluated the use of amiodarone in a predominantly though not exclusively ischemic cardiomyopathy population with  ≥ 10 PVCs /hr. Although the overall study showed no mortality benefit of amiodarone therapy, subgroup analysis of the 193 non-ischemic participants demonstrated a trend towards reduction in all cause mortality [24], with a P value of 0.07. One of the intriguing findings of this randomized, double blind trial was that arrhythmia suppression with amiodarone had no effect on survival.

The Cardiomyopathy Trial (CAT) randomized 104 patients with NIDCM to ICD placement versus optimal medical management and followed them for greater than five years (5.5 ± 2.2 years). ICD therapy had no significant benefit in comparison to medical therapy [25]. The trial may have been underpowered to detect a survival benefit, as the overall mortality did not reach the anticipated 30% in the control group.

The Amiodarone Versus Implantable Cardioverter-Defibrillator in patients with non-ischemic dilated cardiomyopathy and asymptomatic nonsustained ventricular tachycardia (AMIOVIRT trial) evaluated empiric amiodarone therapy vs. prophylactic ICD implantation in 103 patients with NIDCM, EF ≤35%, and asymptomatic NSVT. The three-year survival rate was 89% in both arms of the study with no significant difference between amiodarone and ICD placement in either survival or quality of life endpoints (the trial was stopped at the first interim analysis). Patients treated with amiodarone, however, showed a trend toward improved arrhythmia-free survival compared to the ICD arm.

These two smaller trials, evaluating optimal medical therapy [25] and amiodarone [4] against prophylactic ICD placement, have suggested medical therapy to be as effective as ICD implantation in preventing all cause mortality in asymptomatic patients with NIDCM and non-sustained ventricular tachycardia (NSVT). A more definitive assessment of the benefit of prophylactic ICD implantation in the NIDCM population was, however, performed in the Defibrillators in Non-ischemic Cardiomyopathy Treatment Evaluation (DEFINITE) trial. DEFINITE compared the use of the ICD with standard oral medical therapy vs. medical therapy only of patients with NIDCM and NSVT. Inclusion criteria for the trial included EF ≤35%, symptomatic heart failure, and documented ventricular ectopy (> 10 PVCs/hour) or NSVT on Holter monitor or telemetry within the last 6 months [26]. The trial randomized 458 patients. Arrhythmia mortality accounted for 33% of deaths. At 2 years, mortality in the medical therapy arm was 13.8%, and was reduced to 8.1 % in the ICD arm (p=0.06). The ICD group had a 74% reduction in arrhythmic deaths (p ≤ 0.05). The trial’s primary endpoint (total mortality) failed to reach statistical significance; however, ICD’s were associated with a significantly lower rate of arrhythmic death, the study’s secondary endpoint. Subgroup analysis found that male patients, EF < 20%, QRS duration > 120 msec, and NYHA Class III received the greatest benefit from ICD implantation. NYHA class III was associated with a 67% risk reduction in all cause mortality (p=0.009). The DEFINITE trial has also been criticized for being underpowered.

The Sudden Cardiac Death in Heart Failure Trial (SCD-HeFT) is a recently published primary prevention trial. It enrolled patients with EF < 36% and NYHA class II or III heart failure [27] into three arms, medical therapy vs. medical therapy plus amiodarone vs. medical therapy plus ICD. The endpoint of the study was all-cause mortality. About half of the 2521 patients enrolled had an underlying diagnosis of NIDCM. The five-year all-cause mortality in the ICD group was 28.9%, compared to 34.1% in the amiodarone group and 35.8% in the placebo group. This resulted in a reduction in five-year all-cause mortality by 23% with ICD use compared with the control group. Amiodarone had no effect on survival. The conclusion is that ICD implantation saves lives (but amiodarone doesn't) in patients with class 2 or 3 heart failure, irrespective of the etiology of heart failure. It is interesting to note that in patients with class III heart failure, irrespective of etiology, ICD implantation was associated with a significant survival advantage when compared to amiodarone but not when compared to placebo. As a result of this large randomized, double-blind trial incorporating a placebo arm, prophylactic ICD implantation in NIDCM patients with a LVEF < 36% and NYHA functional class II or III seems justified and risk stratification in this group of patients may no longer be required. Other patient groups (eg. LVEF 36-40% or NYHA class I functional status) will, however, still need risk stratification.

## Risk Stratification

Based on the primary prevention trials there seems to be still some controversy regarding improved survival with ICD therapy in patients with NIDCM. It is believed that benefit would be more readily achievable if a higher risk subgroup could be selected instead of allocating this expensive therapy to the broad population of patients with NIDCM. A number of diagnostic methods have been used to risk stratify patients with cardiomyopathy for elevated risk for sudden death. History of syncope [28-30], Electrocardiographic monitoring [31-36], programmed electrical stimulation (PES) [37-56], signal-averaged ECG (SAECG) [56-58], heart rate variability [48-59], QT dispersion [46], and baroreceptor sensitivity testing [60], heart rate turbulence [63-65] have all been evaluated.

## Syncope

Syncope may be a harbinger of SCD. Comparisons of patients without documented VT/VF but with a history of unexplained syncope and patients who have survived documented cardiac arrest reveal similar mortality [29]. In one particular trial 23 patients with NIDCM and syncope were compared to a similar group of 201 patients that did not have syncope [28]. This trial was non-randomized, with more frequent use of amiodarone in the syncope group (p < 0.04). During the follow-up, there was no statistical difference in mortality between the two groups. However, 83% of the mortality in the syncope group was due to sudden death, compared to 32% in the control group (P < 0.025).

Knight et al. prospectively followed 14 patients who presented with unexplained syncope and who were diagnosed with NIDCM, and had negative electrophysiology study [29] along with a control group of 19 patients with NIDCM who sustained a cardiac arrest.  All patients in both arms of the study received an ICD. During the 24 ± 13 months of follow-up, 50% of patients with unexplained syncope received appropriate ICD shocks in comparison to 42% of the patients who received an ICD for cardiac arrest (P=0.1). Time to first appropriate ICD shock actually occurred earlier in the syncope patients as compared to those with cardiac arrest (32±7 vs. 72±12 months, p =0.01). Due to the small number of patients in this study, the results were not statistically significant. However, the trend suggests that syncope is a marker for arrhythmic death in patients with NIDCM.

Fonarow et al. evaluated 147 patients with NIDCM, syncope, and severely decreased left ventricular function [30]. Twenty-five patients underwent ICD implantation and were compared to 122 controls that were treated medically. The 2-year survival was 84.9% in the ICD group compared to 66.9% in the medically treated group (P=0.04). While this trial was non-randomized, the ICD group had significantly improved 2-year survival although they had more frequent ventricular arrhythmias during monitoring (56% vs. 15% P=0.0001).

These studies suggest that unexplained syncope in patients with NIDCM is a grave prognostic indicator with a significant proportion benefiting from ICD implantation; however that is not to imply that absence of syncope confers any protection from sudden death.

## Electrocardiographic Monitoring

The prognostic influence of electrocardiographic monitoring for the presence of ventricular arrhythmias has been debated for nearly two decades. The incidence of NSVT in patients with NIDCM varies from 33 to 79% [31-32]. Survival in such patients at one year is 92%, and decreases to 88% at two years [4]. Huang et al. followed 35 patients with recently diagnosed NIDCM that had ambulatory electrocardiographic monitoring to evaluate for ventricular arrhythmias [33]. At baseline, 83% had frequent PVCs, 93% had complex PVCs and 60% of patients had NSVT. During follow-up of 34 ± 17 months there were 4 deaths, two of which were sudden. Of the two patients who suffered sudden death during follow-up, one had no evidence of ventricular arrhythmias at baseline. While this study showed a high incidence of NSVT in the NIDCM population, it was not powered to show any correlation between NSVT and sudden death.

Olshausen et al. followed a group of 73 patients with NIDCM for a minimum of 3 years [34]. All patients received ambulatory holter monitoring at baseline. During follow-up, 38% of patients expired. The deaths were evenly divided between those that died of pump failure and those that died due to sudden death.  NSVT was a risk factor for death due to pump failure, but was not for sudden death. On the other hand, Becker et al. followed 256 patients with NIDC [35]. Of those, 99 patients had documented asymptomatic NSVT, while 157 controls with NIDCM were free of documented ventricular arrhythmias. During the 22 ± 14 months of follow-up, both overall mortality and mortality due to sudden death were higher in patients with NSVT than those without arrhythmias (34.2 vs. 9.8%, P=0.0001 and 15.8 vs. 3.7%, P=0.0037).

Grimm et al. recently assessed a cohort of 343 patients with NIDCM using multiple diagnostic tests to assess risk for sudden death [36]. During the 52 ± 21 months of follow-up, 13% of patients had a major arrhythmic event (sustained VT, VF, or sudden death). Ejection fraction was found to be the only significant predictor of a major arrhythmic event, with a relative risk reduction of 2.3 for every 10% increase in ejection fraction (95% CI, 1.5 to 3.3, p=0.0001). The finding of NSVT on holter monitoring was associated with a trend towards an increased risk of major arrhythmic events (RR 1.7, 95% CI 0.9 to 3.3, p=0.11).

The prognostic value of NSVT or frequent ventricular arrhythmias remains unclear. The weight of evidence suggest that it is predominantly a marker of risk for all cause mortality but is not very effective in selecting a high risk group for sudden arrhythmic death.

## Electrophysiologic Testing

Programmed electrical stimulation (PES) has been evaluated in NIDCM. The studies performed were often non-randomized and had small numbers of patients (Table 1). In addition, the variability of stimulation protocols makes assessment of predictive values difficult. Sustained ventricular arrhythmias (monomorphic and polymorphic VT, or VF) may be induced in up to 38% of patients with NIDCM undergoing PES, but this may have limited clinical significance [37].

The reproducibility of programmed stimulation is greater in patient’s who presented with clinical VT as opposed to those with ventricular fibrillation. Milner et al. performed programmed stimulation in 19 patients with NIDCM and symptomatic ventricular arrhythmias; 10 had survived an out-of-hospital cardiac arrest, 8 had monomorphic VT (MMVT), and 1 had NSVT [38]. The mean ejection fraction in this group was 26 ± 9%. 13 (68%) of these patients had their clinical arrhythmia reproduced in the EP lab. The most frequently reproduced rhythm was MMVT (92%), while VF was reproducible in only one patient (8%). Monomorphic VT induced with programmed stimulation does appear to predict future occurrence of spontaneous monomorphic VT of a similar rate and configuration [38-39].

Nevertheless, the diagnostic yield of PES in the NIDCM population has been poor. Stamato et al. performed PES in 15 patients with NIDCM and heart failure [40]. The mean EF in this group was 17%, and they all had NSVT on cardiac monitoring.  PES in this group failed to produce a sustained ventricular arrhythmia in any patients.  Similarly, Das et al. performed PES in 24 patients with NIDCM and a mean ejection fraction of 25 ± 12%. While ventricular arrhythmias were induced in 42% of patients, MMVT was seen in only 8% of the group.

Furthermore the specificity of induced arrhythmias has been called into question. The most common finding with PES in patients with NIDCM is inducible polymorphic VT or VF [37,39,41]. Meinertz et al. evaluated 42 patients with NIDCM and performed PES in all patients [41]. 86% of the study group had polymorphic ventricular arrhythmias, with three or more beats of PMVT induced in 26% of the study group. During the 16 ± 7 months of follow-up, 5% of patients died of sudden death. None of the patients who suffered sudden death had an induced arrhythmia during PES. This suggests that the positive predictive value of induced polymorphic ventricular arrhythmias is low in the NIDCM population.

The use of PES for risk stratification in the NIDCM population has not provided satisfying results. Aside from the rare occasion of inducible MMVT, PES may frequently result in the induction of non-specific arrhythmias. The negative predictive value of a normal study is poor as well.

## Signal Averaged ECG

Several groups have evaluated the performance of the signal averaged ECG (SAECG) to stratify risk for SCD. Many of these studies did not differentiate between CAD and non-ischemic causes of cardiomyopathy, and were not powered for mortality in the NIDCM population. Galinier et al. prospectively followed 151 patients with CHF, 48% of which were diagnosed with NIDCM [42]. At baseline, late potentials were detected in 32.5% of the total patient population. 34% of the NIDCM patients were found to have late potentials (similar to the incidence of late potentials in patient’s with CAD). Late potentials were not found to be predictive of overall mortality or sudden cardiac death but did improve risk stratification for sustained ventricular tachycardia.

In a group of NIDCM patients, Fauchier et al. performed SAECG in 131 patients with mean ejection fraction of 33 ± 12% and followed them for 54 ± 41 months[43]. Late potentials were present in 27% of the patients. During follow-up, 15% of the patients had major arrhythmic events. Patients with late potentials were found to be at increased risk of all-cause cardiac death (RR 3.3, 95% CI 1.5 to 7.5, P = 0.004) and of arrhythmic events (RR 7.2, 95% CI 2.6 to 19.4, P = 0.0001), but not sudden death.

The studies of SAECG in the NIDCM population are complicated by the inclusion of individuals with bundle branch blocks (BBB), which compromises the utility of the SAECG as an adequate method for risk stratification in this population. Mancini et al. evaluated 114 patients with NIDCM with SAECG [44].  In the first year of follow-up, survival was 95% in patients with a normal SAECG, 88% in patients with a BBB, and only 39% in patients with an abnormal SAECG (P < 0.001). During follow-up, of the 20 patients with an abnormal SAECG, 5 (20%) suffered a sudden death, and three others either died from progressive heart failure or required urgent cardiac transplantation. This suggests that an abnormal SAECG confers an increased risk for an arrhythmic episode within a relatively short follow-up period. This study was flawed, however, because of the non-uniform implantation of ICDs in the study group, with patients with BBB having significantly more ICDs placed at baseline (P < 0.05).

An abnormal result on SAECG may be a marker of increased risk of sustained ventricular tachycardia or death [43,44]. As such, the SAECG appears to have an excellent negative predictive value, with the caveat that the presence of bundle branch block may significantly decrease the specificity of SAECG [45]. However, the poor positive predictive value for arrhythmic events and decreased specificity in the significant number of patients with bundle branch block lessens the value of this test.

## QT Dispersion

QT dispersion (QTd) has been studied in the risk stratification of SCD in patients with NIDCM. Few studies have been done, and the results have been unfavorable. Grim et al. performed QT dispersion and related measures in 107 patients with NIDCM and 100 controls without structural heart disease and followed them for 13 ± 7 months [46]. During follow-up, 11% of patients with NIDCM had an arrhythmic event, defined as sustained VT, VF, or sudden death. QT dispersion was noted to be increased in patients with arrhythmic events compared to patients without an arrhythmia during follow-up (76 ± 17 vs 60 ± 26 ms, P=0.03). On the other hand, corrected QT dispersion (QTc) and adjusted QTc were not statistically different between those with and those without arrhythmias during follow-up (80 ± 21 vs 75 ± 35 ms, and 27 ± 6 vs 24 ± 10 ms). This modality is limited due to the overlap between groups with positive and negative results. The Marburg Cardiomyopathy Study [36], confirmed that QT dispersion does not significantly risk stratify patients for sudden death.

## HR Variability

Measures of autonomic function have been evaluated to assess risk for SCD. Heart rate variability is an indirect measure of autonomic tone. Previous studies have shown that heart rate variability (HRV) is predictive of arrhythmic events following myocardial infarction (Table 2). The ATRAMI (Autonomic Tone and Reflexes After Myocardial Infarction) study showed that both decreased HRV and baro-receptor sensitivity, another measure of autonomic tone, are associated with worse outcomes in patients after myocardial infarction [47].

HRV was evaluated in 70 patients with NIDCM and prophylactic ICD implantation. Patients with sustained ventricular arrhythmias as recorded by their defibrillator had similar HRV to those who had no arrhythmic events [48].  Other trials have shown that in patients with NIDCM, HRV was predictive of cardiac death but not ventricular arrhythmias [49,50]. Patients with NIDCM have decreased heart rate variability. However, this decrease is associated with systolic ventricular function, and does not correlate with increased risk of ventricular arrhythmias [49]. The weight of the evidence suggests that due to the poor positive predictive value of HRV, this test has no role in the risk stratification for sudden death of patients with NIDCM [48-50].

## Baroreceptor Sensitivity Testing

Studies of baroreceptor sensitivity (BRS) in the NIDCM population have not focused on risk stratification for sudden death until recently [36]. Menz et al. compared BRS against heart rate variability in a series of 179 patients including those with CAD (27%) and NIDCM (73%) [51]. This trial was designed to determine if there were any differences in these two diagnostic tests in a baseline group of patients with decreased EF.  It was not designed for assessment of prognostic value. Baroreceptor sensitivity was comparable in patients with CAD and NIDCM (6.1 ± 3 vs 6.9 ± 5 ms/mmHg, P=NS).

Grimm, et al. performed an exhaustive trial of risk stratification techniques in patients with NIDCM, including BRS [36]. 343 patients with NIDCM underwent BRS testing, with 26 patients (10%) being excluded due to insufficient response to phenylephrine during BRS testing. No correlation was noted between BRS results and sudden death during the 52 ± 21 months of follow-up. The evidence to date suggests that BRS is not a reliable risk stratifier for sudden death in NIDCM, and may not have additional benefit over other markers of autonomic tone, such as heart rate variability.

## Microvolt T Wave Alternans

Microvolt T Wave Alternans (TWA) is due to beat-to-beat alterations in cellular repolarization due to changes in action potential duration. Kitamura et al described an increased risk of SCD or sustained VT/VF in patients with a history of NIDCM with occurrence of microvolt level TWA at sufficiently low heart rate [52]. Onset of TWA with heart rates below 100/minute conferred increased risk of SCD or sustained VT/VF, with a predictive accuracy of 78% [52]. The onset HR of patients with TWA is independent of the more standard risk factors, including LV ejection fraction, gender, systolic or diastolic BP, or NYHA functional class. This makes it attractive in risk stratifying patients with NIDCM. Unfortunately TWA can be indeterminate in as many as 20% of patients with NIDCM [52,53]. Combining it with the presence of late potentials on SAECG [54] may increase the sensitivity of TWA.

A recent study by Hohnloser et al looked at 137 patients with NIDCM referred to a single center for diagnosis or management of heart failure or evaluation of symptomatic arrhythmias [55].  The follow-up was 14 ± 6 months, with endpoints defined as sudden death and hemodynamically unstable VT or VF. 37 patients in the study had prior ICD implantation. TWA at heart rates ≤110/minutes had a positive predictive value of 22% ± 5% and a negative predicative value of 94% ± 4%. The strong negative predictive value of sustained TWA demonstrated in this study at heart rates <110 bpm suggests a role of TWA testing in risk stratification of patients with NIDCM (Table 3). Further studies to look at larger, more diverse populations, long-term follow-up, and the benefits of serial TWA measurements will clarify the role of this test in risk stratification of patients with NIDCM.

## Heart Rate Turbulence

Heart rate turbulence is an emerging risk profiling method in patients with heart disease [63-65]. It is a method which is based on the electrical property of the heart after a ventricular ectopic beat and can be measured from a 12 lead electrocardiogram or holter monitoring. Immediately post ventricular ectopic beat the native ventricular response accelerates (due to baroreflex activity because of decreased cardiac output) and followed by a slow deceleration back to normalcy. Measurement of these variables when studied along with other risk factors has shown superior risk prediction for sudden cardiac death. This method however will need to be validated with prospective clinical studies.

## Conclusion

As the treatment of heart failure from Non-ischemic dilated cardiomyopathy CHF becomes more refined, it is expected that death from pump failure will be delayed, and risk stratification of these patients for arrhythmic death will become more important. Proper risk stratification based on symptoms, signs, and judicious use of noninvasive and invasive testing is important to determine the subgroup of patients that would benefit most from ICD implantation.

The most effective treatment and prophylaxis for SCD is implantation of an ICD. Due to the considerable cost considerations of these devices it would probably be uneconomical to place these devices in all patients with NIDCM. While it may be expected that the price of these devices individually will decrease over time, due to the increasing number of expected implantations the overall cost to the healthcare system is not likely to diminish. More over majority of the health systems cannot afford these expensive devices on a “blanket” basis. When compared to similar patients with ischemic cardiomyopathy, it appears that the non-ischemic dilated cardiomyopathy patient has a less certain clinical course in terms of sudden cardiac death.  Most of the current trials of risk stratification for patients with NIDCM, with the exception of the SCD-HeFT trial, are underpowered and non-randomized. Clearly further studies that define the precise role of various risk stratification modalities need to be performed. Current indications for ICD implantation miss a fairly large number of patients at risk for sudden death. Appropriate primary prevention and secondary prevention of cardiac arrest in this population will require effective risk stratification to allow delivery of life saving therapy in an economically reasonable fashion.

## Figures and Tables

**Table 1:**
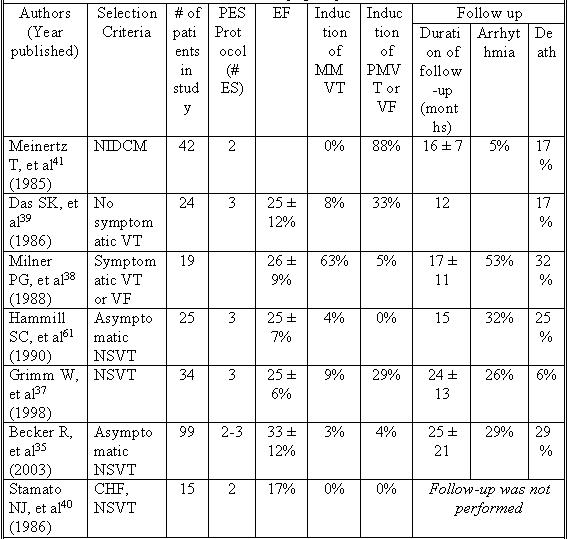
Programmed Stimulation For Risk Stratification in Non-ischemic Dilated Cardiomyopathy

CHF = congestive heart failure, EF = ejection fraction, # ES = number of extra stimuli, MMVT = Monomorphic ventricular tachycardia, NIDCM = Non-ischemic dilated cardiomyopathy, NSVT =Nonsustained ventricular tachycardia, PES = programmed electrical stimulation, PMVT = polymorphic ventricular tachycardia, VF = ventricular fibrillation, VT = ventricular tachycardia

**Table 2:**
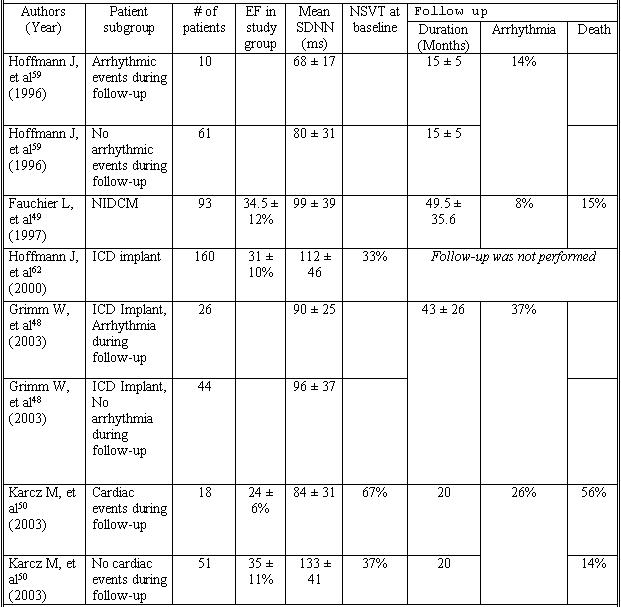
Heart Rate Variability For Risk Stratification in Non-ischemic Dilated  Cardiomyopathy

EF = ejection fraction, NIDCM = Non-ischemic dilated cardiomyopathy, NSVT = nonsustained ventricular tachycardia, Mean SDNN = standard deviation of the mean of normal RR intervals

**Table 3:**
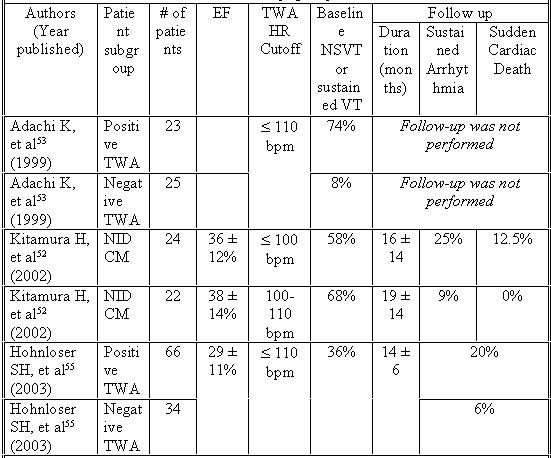
T Wave Alternans For Risk Stratification in Non-ischemic Dilated Cardiomyopathy

All studies used TWA criteria that included alternans voltage  ≥ 1.9 μV and alternans ratio  ≥ 3. Unless specified, events in follow up columns reflect patients with a positive TWA test. EF = ejection fraction, HR = heart rate, NSVT = nonsustained ventricular tachycardia, TWA = T Wave Alternans, VT = ventricular tachycardia.
